# Phytochemical treatments target kynurenine pathway induced oxidative stress

**DOI:** 10.1080/13510002.2017.1343223

**Published:** 2017-06-26

**Authors:** K. Parasram

**Affiliations:** Department of Biology, University of Windsor, Windsor, Canada

**Keywords:** Neurodegenerative diseases, phytochemicals, quinolinic acid, kynurenine, Huntington’s disease, free radicals, oxidative stress, antioxidant

## Abstract

**Objective:** The objective of this paper was to link the phytochemical and metabolic research treating quinolinic acid induced oxidative stress in neurodegenerative disorders.

**Methods:** Quinolinic acid, a metabolite of the kynurenine pathway of tryptophan catabolism, plays a role in the oxidative stress associated with many neurological disorders and is used to simulate disorders such as Parkinson’s disease.

**Results:** In these models, phytochemicals have been shown to reduce striatal lesion size, reduce inflammation and prevent lipid peroxidation caused by quinolinic acid.

**Conclusion:** These results suggest that phenolic compounds, a class of phytochemicals, including flavonoids and diarylheptanoids, should be further studied to develop new treatments for oxidative stress related neurological disorders.

## Abbreviations

ERK: extracellular signal-regulated kinase
RAGE: receptor for advanced glycation end products
NAD+: nicotinamide adenine dinucleotide
NFκβ: nuclear factor kappa-light-chain-enhancer of activated B cells

## Introduction

Neurodegenerative disorders affect hundreds of millions worldwide [1]. Neurological disorders range from depression to autoimmune disorders, such as amyotrophic lateral sclerosis, and while frequently undiagnosed at an early stage, these diseases can quickly progress into restriction of movement, cognition and altered emotional state [2]. Causes of neurological disorders include alteration in metabolic pathways, protein structure, or physical damage to the brain [3]. Current therapies for neurological disorders have limited effectiveness and drive alternative approaches – such as naturopathic medicine [4,5].

Huntington’s disease, Alzheimer’s disease and many other neurological disorders have been linked to oxidative stress, which refers to damage to the structure of biomolecules due to reactive oxygen species, such as hydrogen peroxide (Table 1) [6–9]. The current treatments for neurodegenerative diseases are primarily symptom-based with few drugs playing preventative roles [4–9]. Phytochemicals, notably phenolic compounds that contain a hydroxyl functional group, are known for their antioxidant, antitumorigenic, and antiviral activities through free-radical scavenging and/or chelating action [10]. This research has quickly expanded using traditional medicine as a guide for novel therapeutics in the treatment of neurological disorders. High throughput analysis techniques such as mass spectrometry, have enabled researchers to profile more plant extracts than ever before, quickly expanding the list of potential lead compounds [11]. The free-radical scavenging ability of phytochemicals is tested using known oxidative agents, such as 3-nitropropionic acid and quinolinic acid, to investigate biological activity in cells [12].

**Table 1.** An overview of neurological disorders focusing on the main affected regions and the link to oxidative stress, increased glutamate levels cause excitotoxicity and may be triggered by quinolinic acid.

Neurological disease	Symptoms	Affected region	Role of oxidative stress	References
Amyotrophic Lateral Sclerosis	Progressive muscle weakness leading to paralysis	Central nervous system (brain stem, spine, cortex) – motor neurons	Mutations in superoxide dismutase 1 sensitizing motor neurons to excitotoxicity, lipid peroxidation, damage to astrocytes and microglia, and inflammation	[2,3]
Multiple Sclerosis	Muscle weakness, shaking	Loss of myelin sheath in the central nervous system	Inflammation	[3]
Huntington’s Disease	Impairment of motor, cognitive, and behavioural function	Central nervous system – cortex, striatum	Inflammation, lipid peroxidation	[3]
Parkinson’s Disease	Muscle weakness, resting tremor, postural instability	Substantia nigra – dopaminergic neurons	Interferes with dopamine metabolism; ROS cause neuron death	[3,4]
Alzheimer’s Disease	Impaired memory and cognitive functions	Amyloid beta plaques and tau protein accumulation in the central nervous system notably the hippocampus	Involved in toxicity associated with amyloid beta plaques, inflammation response of microglia, and lipid peroxidation	[3,5,6]

ROS: reactive oxygen species [2–6].

**Figure 1.**
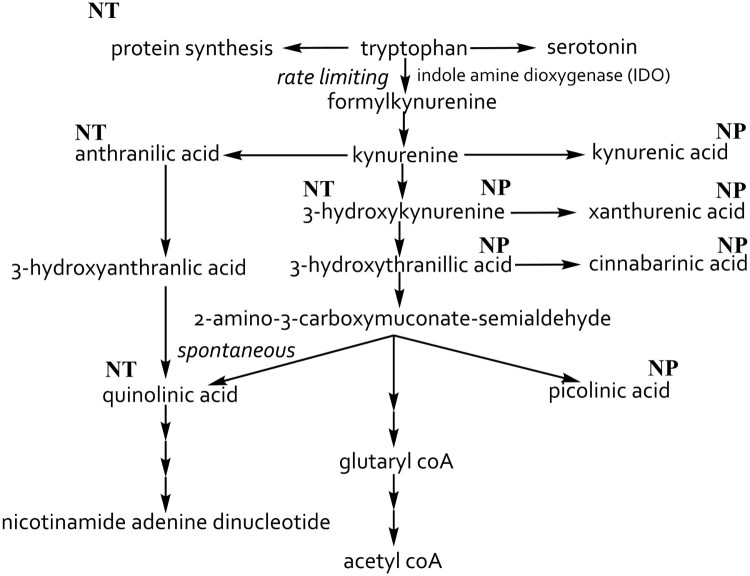
The kynurenine pathway of tryptophan metabolism. Tryptophan is transported into the brain by the l-amino acid transporter and converted by indole amine dioxygenases (IDO), rate limiting step, into formylkynurenine and then by kynurenine formamidase into kynurenine. Kynurenine can then be either converted into 3-hydroxykynurenine by kynurenine hydroxylase then into 3-hydroxythranillic acid. Once 3-hydroxythranillic acid is produced it can then be converted to quinolinic acid spontaneously or picolinic acid by picolinic carboxylase. 3-hydroxylkynurenine can be converted to xanthurenic acid and kynurenine into kynurenic acid by kynurenine aminotransferases. NP: neuroprotective; NT: neurotoxic [2,6,22,25,38].

When studying neurological disorders the kynurenine pathway of tryptophan metabolism (Figure 1) is of particular interest since it has been correlated with many neurological disorders, such as depression and epilepsy [13–16]. The oxidative agent quinolinic acid is linked to Huntington’s disease, amyotrophic lateral sclerosis, suicide, aggression and other disorders [2,14,17–20]. The kynurenine pathway, which occurs in most cells of the central nervous system, catabolizes tryptophan into quinolinic acid, an *N*-acetyl-d-aspartate (NMDA) receptor agonist and precursor of nicotinamide adenine dinucleotide (NAD+), and picolinic acid via kynurenine [2,21]. Tryptophan is also involved in protein synthesis and is a precursor for serotonin [22]. Kynurenines, metabolites of the kynurenine pathway, often have pro and antioxidant properties with the aromatic hydroxyl acting as an electron acceptor [22].

Quinolinic acid, an excitatory kynurenine antagonized by kynurenic acid, is a choline acetyl transferase known to produce neuronal lesions in the cerebrum at low concentrations [17,23]. Quinolinic acid and kynurenic acid form an important balance in maintaining levels of oxidative stress [24]. Quinolinic acid’s neurotoxicity occurs through three methods: increased glutamate signalling, acting as an *N*-methyl-d-aspartic acid receptor agonist, or lipid peroxidation [19]. Picolinic acid, which is produced from 2-amino-4-carboxymuconic-6-semialdehyde in the presence of picolinic carboxylase instead of spontaneously transforming into quinolinic acid, acts as an endogenous neuroprotective agent by chelating iron, necessary for quinolinic acid to function, or zinc to antagonize quinolinic acid [19,25]. Therefore, the balance of the kynurenine pathway metabolites is necessary to maintain normal brain function.

Since the kynurenine pathway metabolites can become sources of oxidative stress they are implicated in many neurological diseases. Metabolites, such as quinolinic acid, are commonly used to test for the neuroprotective potential of phytochemicals. This paper will highlight recent research that indicates the potential for the use of phytochemicals as therapeutic neuroprotective agents by examining phytochemicals showing neuroprotective behaviour and resistance to quinolinic acid induced oxidative stress. The research articles referenced in this paper were selected for their study of neurological disorders, use of kynurenines to induce oxidative stress, and testing of phytochemicals to treat oxidation-induced damage.

## Phytochemical protection

In a Parkinson’s cell model, *Olea europaea,* which contains, a phenolic compound called oleuropein, resisted oxidative stress caused by 6-hydroxydopamine and prevented neuronal death showing a decrease in neuronal death, Bax and Bcl-2 as apoptotic markers and superoxide (*p* < 0.05) [26]. Similarly, *Ficus religiosa* extract treated rats showed improved behaviour and reduced lipid peroxidation when treated with 6-hydroxydopamine [4]. *Fructus mume* extract, (200 mg/kg oral), decreased p-ERK, astrocyte cell numbers and pro-inflammatory cytokines in the hippocampus by modulating RAGE signalling white matter is promising for the treatment of vascular dementia, as modelled by chronic cerebral hypoperfusion in Wistar rats [27]. Eugenol, found in cloves, was tested in SHSY5Y cells in diabetes and, hyperglycemic, conditions to assess its potential as a treatment for neuro-oxidative problems in diabetic patients [28]. The results show that eugenol treated cells have decreased oxidative markers and increased glutathione (GSH) levels [28].

Huntington’s Disease is characterized by striatal neuronal degeneration that may be due to the aggregation of mutant huntingtin protein [29]. Oxidative stress is evident in Huntington’s disease in the reduction of Complex II/III activity in the mitochondrial electron transport chain, superoxide dismutase I and glutathione peroxidase [30]. 3-nitropropionic acid causes mitochondrial dysfunction by disrupting oxidative phosphorylation and the electron transport chain due to its inhibition of succinate dehydrogenase and mitochondrial complex II thereby causing a Huntington’s phenotype [30,31]. Embelin, from *Embelia ribes*, was tested against 3-nitropropionic acid oxidative damage in adult Wistar rats and showed that when embelin, 10 or 20 mg/kg/day oral, was administered with 3-nitropropionic, 15 mg/kg/day intra-peritoneal, after 8 days of damage by 3-nitropropionic acid, there was a reversal of striatal neuronal damage, and a decrease in brain lesion area >69% (*p* < 0.001), improved behaviour and decreased oxidative stress [29].

## Phytochemicals and quinolinic acid

In the kynurenine pathway, the conversion of tryptophan into formylkynurenine is catalyzed by indoleamine 2,3-dioxygenase and is the rate limiting step of the pathway. Consequently, its inhibition imparts significant neuroprotective effects by controlling quinolinic acid production [32]. Ferulic acid, found in wheat bran and rice was shown to inhibit indoleamine 2,3-dioxynease activity by inhibiting mitogen-activated protein kinase (MAPK) and NFκβ signalling in lipopolysaccharide activated microglia [32].

*Boerhaavia diffusa* extract was tested in rat brain homogenates to assess its ability to negate oxidative damage caused by thiobarbituric acid reactive substances including 3-nitropropionic acid and quinolinic acid [12]. This extract decreased the thiobarbituric acid reactive substances and lipid peroxidation while increasing antioxidant, superoxide dismutase and reduced glutathione, concentrations (*p* < 0.05) [12]. *Crassocephalum crepidiodes* hydrophilic extract was shown to inhibit acetylcholinesterase activity and exhibit free-radical scavenging activity (*p* < 0.05) as well as potential for inhibition of lipid peroxidation (*p* > 0.05) [33].

Examination of the neuroprotective potential of *Centella asiatica* or its butanolic, ethyl acetate or dichloromethane extracts inhibited lipid peroxidation and thiol oxidation in the striatum, hippocampus and cerebral cortex (*p* < 0.05) [34]. *Clerodendrum volubile*, a traditional treatment for neurological disorders, was tested to determine its phenolic composition and free-radical scavenging activity. Leaf extracts reduce malondialdehyde oxidase levels, lipid peroxidation and monoamine oxidase activity (*p* < 0.05) showing its potential as a neuroprotectant [35]. The flavonoids catechin and epicatechin found in cocoa were tested in rat striatal slices treated with quinolinic acid restored GSH levels, protecting against oxidative damage [10].

When quinolinic acid was used to induce neuroinflammation in rats, curcumin and curcumin in combination with piperine improved motor performance (grip strength and narrow beam walk) and prevented neurodegeneration in the striatum [36]. Rats on a fatty-acid-rich diet, from fish and olive oil, had reduced oxidative damage in the striatum, normal circling behaviour and increased GABA levels, when microinjected with quinolinic acid [37]. Thus, demonstrating the neuroprotective nature of fatty acid free-radical scavenging activity [37].

## Conclusion

Oxidative stress of the central nervous system can be caused by metabolites of the kynurenine pathway, such as quinolinic acid, initiating inflammatory responses that are associated with many neurological disorders. Plants are widely used as rich sources of new chemicals for disease treatment due to the biological activity of phenolic compounds, a class of phytochemicals, including flavonoids and diarylheptanoids, often associated with antioxidant and anti-inflammatory activity. This property can be harnessed to treat neurological disorders that are associated with oxidative damage. Further research is required to elucidate the mechanism(s) of action, specifically the interaction between phytochemicals and reactive oxygen species.
